# Aging Behaviors of Phenol-Formaldehyde Resin Modified by Bio-Oil under Five Aging Conditions

**DOI:** 10.3390/polym14071352

**Published:** 2022-03-26

**Authors:** Yuxiang Yu, Chao Li, Chenxin Jiang, Jianmin Chang, Danni Shen

**Affiliations:** 1 Laboratory of Material Innovation Design and Intelligent Interaction, Zhejiang Sci-Tech University, 928 Seconded Avenue, Xiasha High Education Zone, Hangzhou 310018, China; zoe_li1989@hotmail.com (C.L.); qq1340100553@163.com (C.J.); dnshen@yeah.net (D.S.); 2 College of Materials Science and Technology, Beijing Forestry University, 35 Qinghua East Road, Haidian District, Beijing 100083, China; cjianmin@bjfu.edu.cn

**Keywords:** aging behaviors, phenol-formaldehyde resin, bio-oil, aging condition

## Abstract

The bio-oil phenol-formaldehyde (BPF) resin, prepared by using bio-oil as a substitute for phenol, has similar bonding strength but lower price to phenol-formaldehyde (PF) resin. As a common adhesive for outdoor wood, the aging performance of BPF resin is particularly important. The variations in mass, bonding strength, microstructure, atomic composition, and chemical structure of BPF resin under five aging conditions (heat treatment, water immersion, UV exposure, hydrothermal treatment, and weatherometer treatment) were characterized by scanning electron microscope, X-ray photoelectron spectroscopy, and Fourier transform infrared spectroscopy, respectively. Compared under five aging conditions, after aging 960 h, the mass loss of plywood and film was largest under hydrothermal treatment; the bonding strength of plywood, the surface roughness, and O/C ratio of the resin film changed most obviously under weatherometer treatment. FT-IR analysis showed that the decreased degree of peak intensity on CH_2_ and C–O–C characteristic peaks of BPF resin were weaker under water immersion, hydrothermal treatment, and weatherometer treatment than those of PF resin. The comparison of data between BPF and PF resins after aging 960 h showed that adding bio-oil could obviously weaken the aging effect of water but slightly enhance that of heat. The results could provide a basis for the aging resistance modification of BPF resin.

## 1. Introduction

Phenol-formaldehyde (PF) resin has been widely used in outdoor wooden products because of its strong mechanical properties, stable molding processing performance, high flame retardancy, etc. [1,2]. Phenol, as the main raw material of PF resin, is derived from petroleum-based chemicals. To reduce the use of phenol, modifiers from renewable natural compounds such as lignin [3,4,5], tannin [6], and cardanol [7] have been a focus of research in recent years.

Bio-oil, a liquid product from the fast pyrolysis of renewable biomass, is rich in phenols, aldehydes, esters, and other organic substances [8,9]. Many researchers have successfully used bio-oil to partially replace phenol to synthesize the bio-oil phenol-formaldehyde (BPF) resin [10,11,12,13,14,15,16,17]. The National Renewable Energy Laboratory [10] first prepared BPF resin by substituting the phenolic substances extracted from bio-oil for 50 wt.% phenol. Aslan et al. [11] used bio-oil to substitute the phenol, and found that the 10 wt.% bio-oil modified PF had a similar molecular structure and higher tensile-shear strength to commercial PF. However, the refining process of bio-oil is too complicated and the solvent recovery is not high, which increased the cost of BPF resin [18,19]. Some researchers began to prepare PF resin with whole bio-oil. Chaouch et al. [12] showed that the BPF whose substitution degree was up to 50 wt.%, presented a nice storage stability and thermal stability, and good shear strength comparable to commercial PF. Dong et al. [13] found that when the replacement ratio of phenol was 20 wt.%, the plywood prepared by BPF resin had better wet shear strength, flexural elastic modulus, and fracture modulus.

As an adhesive for outdoor wooden products, aging property is quite important for BPF resin [20,21]. The aging of BPF resin highly depends on its molecular structure [22]. For example, the phenolic hydroxyl and methylene groups in the molecular chain of BPF resin are easy to be oxidized [23]. The numbers of hydrophilic phenol hydroxyl and hydroxymethyl groups of BPF resin easily absorb atmospheric moisture, resulting in the damage of the glue line and hydrolysis of resin [24,25].

However, at present, studies on BPF resin mainly focus on the synthesis process and basic performance characterization. To the best of our knowledge, little literature seems to be available on the aging properties of BPF resin. In this study, BPF resin was treated under five aging conditions (heat treatment, water immersion, UV exposure, hydrothermal treatment, and weatherometer treatment). The variations in the mass and bonding strength of BPF resin under the five aging conditions were examined. Furthermore, the changes of microstructure, atomic composition, and chemical structure of BPF resin under five aging conditions were characterized to understand the impact mechanism of adding bio-oil to PF resin.

## 2. Materials and Methods

### 2.1. Materials

Bio-oil, an acid liquid (pH 3.5), was obtained by fast pyrolysis of *Larix gmelinii* (Rupr.) Kuzen in a fluidized bed at 550 °C for 2–3 s by the Lab of Fast Pyrolysis of Biomass and Productive Utilization (Beijing Forestry University, Beijing, China). The bio-oil was composed of 23.2% phenols, 20.8% ketones, 9.0% aldehydes, 6.0% organic acids, 29.8% water, and 4.1% other compounds. Phenol, formaldehyde (aqueous solution, 37 wt.%), and sodium hydroxide (NaOH) were purchased from Xilong Chemical Reagent Co., Ltd., Guangdong, China. Poplar veneers (400 mm × 400 mm × 1.5 mm, 8% moisture content) were provided by Xinda wooden Co., Ltd., Hebei, China.

### 2.2. Synthesis and Characterization of Resins

The BPF resin (20 wt.% substitute rate of bio-oil to phenol) was synthesized and characterized according to Yu [26]. The molar ratio of phenol (including bio-oil)/formaldehyde/NaOH was 1:2:0.5. A control reference sample with pure phenol (0 wt.% bio-oil), denoted as PF resin, was also prepared. The characteristics of prepared resins are listed in Table 1.

### 2.3. Aging Tests

The aging specimens (three-layer plywood (100 mm × 25 mm × 3 mm) and resin films (80 mm × 10 mm × 2 mm)) were prepared according to Yu [27]. Then, the aging specimens were treated under five aging conditions: (1) heat treatment (60 °C); (2) water immersion (25 °C distilled water); (3) UV exposure (UVA-340, 40 W); (4) hydrothermal treatment (45 °C and 95% relative humidity); and (5) weatherometer treatment (Yiheng Co., Ltd., Shanghai, China) according to the ASTM G 154. Each 12 h weathering cycle consisted of 8 h of UV (UVA-340, 40 W) exposure at 60 °C and 4 h condensation at 50 °C. The plywood specimen number was 12, and the resin film specimen number was 6.

### 2.4. Analysis

The viscosity and pH of the resin were measured with NDJ-5S rotating viscometer (Shanghai, China) and pH S400-B (Mettler Toledo, Switzerland), respectively. The solid content and water absorption of resin were tested by China National Standards (GB/T 14074-2013) and GB/T 1034-2008, respectively. The bonding strength of the plywood was evaluated on the basis of Chinese standard (GB/T 17657-2013). In this test, 12 plywood specimens with dimension of 25 mm × 10 mm were dipped into boiling water for 4 h, dried in 60 ± 3 °C for 20 h, and dipped into boiling water for 4 h, then submersed in cold water for 1 h. Then, the samples were tested with a speed at 10.0 mm/min of the cross head.

In the test of mass loss, 10 specimens of plywood and resin films were placed in a drying oven at 108 ± 3 °C for 24 h and cooled to room temperature in a dryer, then weighed (*m*_1_). Then, the specimens were treated under five aging conditions. After aging, the specimens were dried and cooled again, and then weighed (*m*_2_). The mass loss (*M_m_*) is calculated by Formula (1).

(1) $$M_{m}=\frac{m_{1}-m_{2}}{m_{1}}\times 100\%$$

where *m*_1_ is the mass before aging and *m*_2_ is the mass after aging.

The scanning electron microscope (SEM) analysis of resin films was measured by SU8010 SEM (Hitachi, Tokyo, Japan) at 5.0 kV accelerating voltage. The X-ray photoelectron spectroscopy (XPS) analysis of the resin films was conducted by PHI Quantera SXM (ULVAC-PHI, Japan) with a 200 μm monochromatic Al X-ray source at 55 eV pass energy, 0.1 eV step size, and 45 degree take-off angle. The Fourier transform infrared spectroscopy (FTIR) analysis of resin films was recorded in a Vertex70 FTIR (Bruker, Karlsruhe, Baden Wurttemberg, Germany) over the range of 400 to 4000 cm^−1^ with a 4 cm^−1^ resolution and 64 scans.

## 3. Results and Discussion

### 3.1. Mass Loss

**Figure 1:**
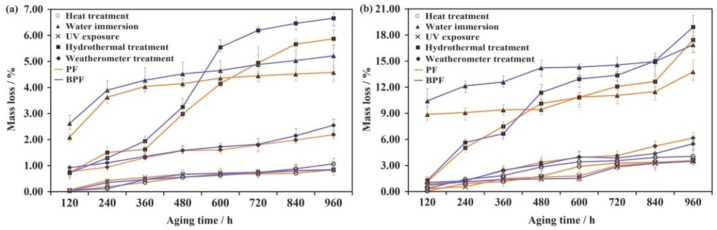
Variations in the mass loss of BPF and PF resins plywood (**a**) and films (**b**) under five aging conditions.

The changes in the mass loss of plywood and films with BPF and PF resins under five aging conditions are shown in Figure 1. As can be seen in Figure 1a, with increasing aging time, the mass loss of plywood increased slowly during heat treatment, which was due to the volatilization of small molecule substances in the plywood. During the water immersion, the mass loss of plywood increased rapidly at first, then slowly as the increasing aging time, which was caused by the dissolving of water-soluble substances in the plywood. The changes on mass loss of plywood under UV exposure were similar to those under heat treatment, owing to the elevated temperature caused by UV light. With the increase of aging time, the mass loss of plywood under hydrothermal treatment increased slowly at first, then increased rapidly. These variations on mass loss under hydrothermal treatment were the combination of heat and water, the initial mass loss was small because water was only serving as a solvent and heat only caused the volatilization of small molecule substances at first. However, over time, the elevated temperature improved the infiltration capacity of water, which increased the damage of water to plywood [28]. This was the reason for the rapid mass loss. The mass loss eventually became stable because the unstable substances had been largely lost in the previous stage.

Compared between the five aging conditions, after 960 h aging, the mass loss under hydrothermal treatment was largest. Moreover, the second largest mass loss happened under water immersion. This suggests that (1) water had a greater influence on the mass loss of plywood than heat and UV light; and (2) the impact on the mass loss of plywood under hydrothermal treatment was stronger than those under heat treatment or water immersion alone. The mass loss of plywood under weatherometer treatment was lower than those under the hydrothermal treatment and water immersion. The possible reason is that the condensation time in the weatherometer treatment only accounted for 1/3 of each cycle, decreasing the impact of water.

In the case of resin films, all five aging conditions resulted in a mass loss (Figure 1b). However, with respect to the plywood, several differences are noticeable: (1) the mass loss of resin films was about 3 times larger than those of plywood, indicating that the film was more easily aged than wood; (2) the mass loss under water immersion lacked the rapid growth stage at first, which meant that water could more easily enter into the resin than wood and had a greater impact on the resin film even in the early aging stage; and (3) under hydrothermal treatment and water immersion, the mass loss of resin film began a rapid increase stage after reaching a slow growth stage. These indicated that the mass loss of resin film in the early stage might be caused by the dissolution of water-soluble substances, and the subsequent mass loss was due to the partial hydrolysis of resin.

As shown in Figure 1, compared to PF resin, the mass loss of BPF resin plywood and films were similar under heat treatment, UV exposure, and weatherometer treatment. However, the differences in mass loss between BPF and PF resins under hydrothermal treatment and water immersion were larger, owing to the great number of small molecule and water-soluble substances in bio-oil.

### 3.2. Bonding Strength

**Figure 2:**
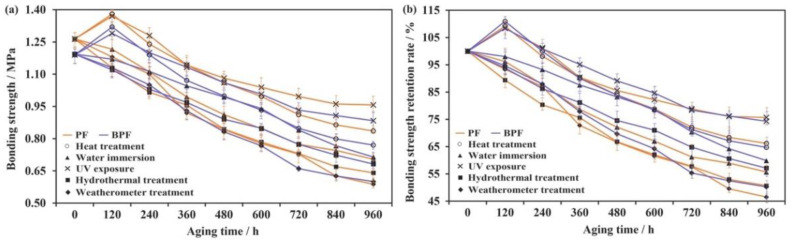
Variation in the bonding strength (**a**) and its retention rate (**b**) of BPF and PF resin plywood under five aging conditions.

The changes in the bonding strength (a) and its retention rate (b) of BPF and PF resin plywood under five aging conditions are displayed in Figure 2. As shown in Figure 2, with increasing aging time, the bonding strength increased at first because of the post curing of resins. High temperature could enhance the movement ability of the polymer chain, which led the unreacted groups to get close to react and cross-link, thus improving the bonding strength [27,29]. Then, the bonding strength began to decrease with increasing aging time owing to the volatilization of small molecule substances, which made the adhesive layer become brittle or even break [30]. Meanwhile, the carbonyl and hydroxyl groups in resin are easy to oxidize under the combined action of high temperature and oxygen, leading to the fracture of chemical bonds in the resin structure [22]. The changes in the bonding strength of plywood under UV exposure were similar to those under heat treatment, owing to the elevated temperature caused by UV light.

As seen in Figure 2, during the water immersion, the bonding strength gradually decreased. The changes under water immersion could be divided into two parts. The first stage is the macroscopic interface penetration stage. Water could gradually infiltrate into the bonding interface to form a weak interface layer, which could destroy the chemical bond, hydrogen bond, and van der Waals forces between adhesive and wood, leading to the decrease of bonding strength [28,31]. The second stage is the microscopic material penetration stage. In this stage, water infiltrated into the adhesive to break the hydrogen bond and other secondary bonds among the adhesive molecules, resulting in the deterioration of bonding strength [31].

With the increase of aging time, the bonding strength of plywood under hydrothermal treatment decreased, and the decrease rate was higher than that during the water immersion and heat treatment. The possible reasons are (1) the destructive effect on the bonding interface from vaporous water was greater than liquid water; (2) the aging effect on the adhesion from water was larger than heat [22]; and (3) high temperature increased the moving speed and infiltration capacity of water. Under weatherometer treatment, the bonding strength of plywood decreased rapidly with increasing aging time. The bonding strength retention rate under weatherometer treatment was lowest because the aging under the weatherometer treatment is the comprehensive aging of water, heat, oxygen, and UV light.

As shown in Figure 2, after aging 960 h under heat treatment, the bonding strength retention (64.71%) of BPF resin plywood was smaller than that (66.14%) of PF resin plywood. This is probably because adding bio-oil reduced the heat resistance of resin. However, under water immersion and hydrothermal treatment, the bonding strength retention (58.89% and 57.13%) of BPF resin plywood was greater than that (55.70% and 40.71%) of PF resin plywood. These figures indicate that the addition of bio-oil could improve the water resistance of resin. The aging effect from UV light was almost the same as BPF and PF resin plywood, but the destructive effect on the bonding interface from the water was bigger than that from heat. This is the reason for the higher bonding strength retention (50.26%) of BPF resin plywood than that (46.53%) of PF resin plywood under weatherometer treatment after aging 960 h.

### 3.3. SEM Analysis

**Figure 3:**
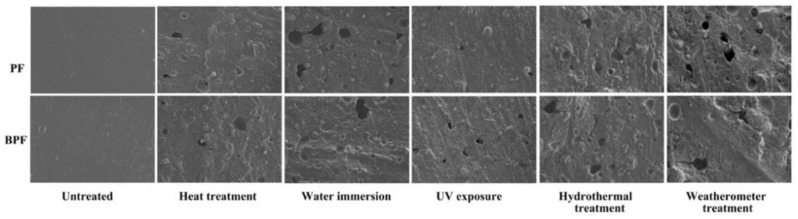
SEM images of BPF and PF resin films under five aging conditions for 960 h (×500).

The SEM images at 500 magnification of BPF and PF resin films under five aging conditions for 960 h are shown in Figure 3. As seen in Figure 3, the surfaces of the unaged BPF and PF resins were very smooth, indicating that the degree of polymerization of BPF resin and PF resin were both good. When aging for 960 h, the roughness of the resin surface under five aging conditions increased significantly, and particles and holes appeared. Comparing among the five aging conditions, it was found that the surface roughness of resin after 960 h aging under weatherometer treatment was the greatest, and the number and diameter of holes were the largest. The second most obvious change of resin surface after aging 960 h was under hydrothermal treatment. This meant that the effect of comprehensive aging on the resin surface was higher than that of single aging. Compared with three single aging tests, the resin surface roughness and holes under water immersion were relatively severe after aging 960 h, indicating that water had a greater impact on the surface roughness of resin film than heat and UV light.

As shown in Figure 3, compared with PF resin, BPF resin exhibited similar surface conditions under high temperature and UV after aging 960 h. In addition, the surface roughness of BPF resin film was slightly bigger than that of PF resin. However, the surface aging degree of BPF resin film was lower than that of PF resin under water immersion, hydrothermal treatment, and weatherometer treatment. One possible reason is that bio-oil improved the water resistance of resin [27]. Another possible reason is that bio-oil improved the toughness of resin, reducing the fracture of resin owing to the stress caused by water swelling [32].

### 3.4. XPS Analysis

**Figure 4:**
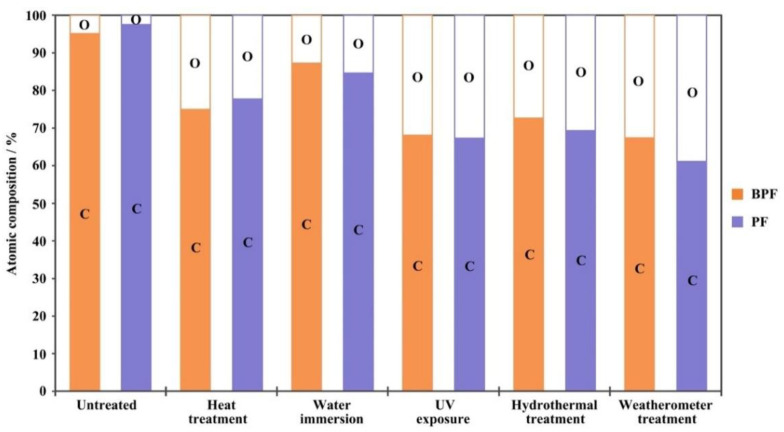
XPS surface atomic composition of BPF and PF resin films under five aging conditions for 960 h.

The XPS surface atomic composition of BPF and PF resin films under five aging conditions for 960 h are shown in Figure 4. It can be seen from the Figure 4 that after the aging treatment, the surface atomic composition of BPF and PF resins changed significantly. After 960 h of aging, the C content of the resin film surface decreased and the O content increased, which means that oxidative aging occurred.

Comparing among the five different aging conditions, it was found that UV exposure had the greatest impact on the oxidation degree of the resin surface. After aging under the UV exposure and weatherometer treatment, the O content of the resin surface increased most obviously. Heat also had a strong influence on the resin surface composition, compared with water immersion. This could be explained by the resin in the water immersion being less exposed to oxygen. After 960 h of aging, the O content under UV exposure was higher than that under hydrothermal exposure, indicating that the combined effect of UV light and heat on the composition of the resin surface was higher than that of water and heat. UV light and heat both had an activating effect on generating free radicals and triggering oxidation reactions during aging.

The changes in surface atomic composition of BPF and PF resin films after aging 960 h under five aging conditions were similar. This means that BPF and PF resin surface have a similar structure. Compared with the PF resin, the initial O/C ratio of the BPF resin film surface was larger, which is due to the abundant oxygen-containing substances in bio-oil. In the comparison of five aging conditions, the O content of the BPF resin film surface was lower than that of the PF resin under water immersion, hydrothermal, and weatherometer treatment after aging 960 h, but higher under heat treatment. The prerequisite for oxidation is contact, and the oxidation degree is related to the contact area. Heat can volatilize the small molecular substances in the resin, and water can dissolve the water-soluble substances in the resin, which will provide a channel for oxygen to enter the resin. SEM analysis (Figure 3) showed that after 960 h of aging, the BPF resin surface was smoother than that of PF resin, except under heat treatment. This might be the reason for the changes in the surface atomic composition of BPF and PF resin films after aging.

### 3.5. FTIR Analysis

**Figure 5:**
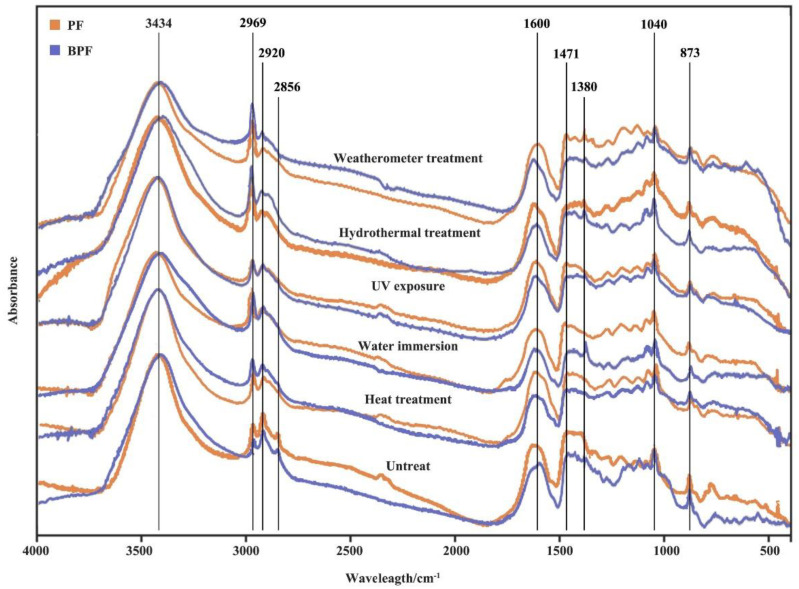
FTIR images of BPF and PF resin films under five aging conditions for 960 h.

The infrared spectrum changes of BPF and PF resin films before and after 960 h aging under five different aging conditions are shown in Figure 5, and their characteristic absorption peaks are shown in Table 2 [11,27,33].

PF resin is mainly a high molecular polymer connected by methylene bridge and ether bond bridge and the content of methylene bridge and ether bond bridge could indirectly represent the polymerization degree of PF resin [10,34]. After aging for 960 h, the stretching vibration peaks of methylene (CH_2_) appeared at 2920 cm^−1^ and 2856 cm^−1^, obviously weakened. The stretching vibration peak of the ether bond (C–O–C) which was connected to the alkane on the benzene ring at 1040 cm^−1^ was also significantly weakened after aging 960 h, indicating that the methylene bridge and ether bond bridge had broken after aging. The characteristic peak at 873 cm^−1^ was the stretching vibration peak outside the benzene ring. After aging, the peak intensity of the C–H (873 cm^−1^) on the benzene ring weakened, indicating that the chemical bonds of resin were broken during aging, and small molecules were formed. The CH_3_ stretching vibration peak at 2969 cm^−1^ increased after aging, which indirectly indicated the increase of small molecules.

Compared among the five aging conditions, the CH_3_ stretching vibration peaks at 2969 cm^−1^ increased more obviously under weatherometer treatment and hydrothermal treatment after aging 960 h, and the CH_2_ stretching vibration peaks at 2930 cm^−1^ and 2850 cm^−1^ were also slightly higher than other aging conditions. This shows that comprehensive aging had a stronger influence on resin molecular structure than single aging. Comparing heat treatment, water immersion, and UV exposure, it was found that after 960 h of aging, the characteristic peak of resin under water immersion changed more obviously, indicating that the influence of water on the resin structure was stronger than that of heat and UV. The result was the same as the changes of bonding strength.

Compared with BPF and PF resin, in heat treatment and UV exposure, the changing degree of peak intensity of CH_2_ and C–O–C characteristic peak were basically the same. However, under water immersion, hydrothermal treatment, and weatherometer treatment, the peak intensity of the characteristic peaks of CH_2_ and C–O–C of BPF resin were weaker than those of PF resin. This might be due to (1) the addition of bio-oil improved the water resistance of the resin, thereby reducing the aging effect of water on the resin; and (2) the addition of bio-oil reduced the pH value of resin (Table 1), thereby reducing the alkalinity of the resin soaking liquid. PF resin was easy to decompose in an alkaline environment, and the decrease of environmental alkalinity could also weaken the aging degree of phenol resin.

## 4. Conclusions

Bio-oil was used as a phenol substitution to synthesize the BPF resin, and as an outdoor adhesive, its aging performance was analyzed under five aging conditions (heat treatment, water immersion, UV exposure, hydrothermal treatment, and weatherometer treatment). Comparing among the five aging conditions, the mass loss of plywood and film with BPF resin was greatest under hydrothermal treatment. The most obvious changes in the bonding strength of plywood, the apparent morphology, and surface O/C radio of the BPF resin film were under weatherometer treatment. This indicates that the effect of water on the aging performance of BPF resin was greater than that of heat and UV light. The comparison of data between BPF and PF resins after aging 960 h showed that adding bio-oil could obviously weaken the aging effect of water but slightly enhance that of heat. These results could provide a basis for the aging resistance modification of BPF resin.

## Figures and Tables

**Table 1:** Characteristics of BPF and PF.

Resins	Characteristics			
	pH (25 °C)	Viscosity (25 °C, mPa·s)	Solid Content (%)	Water Absorption (25 °C, 24 h, %)
PF	10.86 ± 0.12	127 ± 25	47.34 ± 0.24	25.78 ± 0.69
BPF	10.34 ± 0.17	327 ± 67	45.52 ± 0.33	32.14 ± 0.38

**Table 2:** Peaks and assignment of FTIR spectra for BPF and PF resins.

Wave Number (cm^−1^)	Vibration Form	Absorption Peak Attribution
3434	ν(–OH) ^a^	Stretching vibration of phenolic hydroxyl and alkyl hydroxyl
2969	ν(CH_3_)	CH_3_ stretching vibration
2920, 2856	ν(CH_2_)	CH_2_ stretching vibration
1600, 1471, 1380	ν(C=C)	Benzene ring skeleton stretching vibration
1040	ν(C–O–C)	CO stretching vibration of aromatic ether connected with alkyl group
873	δ(C–H) ^a^	C—H out-of-plane bending vibration when benzene ring (1,2,4 or 1,4) is substituted

^a^ ν: Stretching vibration; δ: Bending vibration.

## Data Availability

Not applicable.
